# Supplementary material for: Fertilization reduces aphid population growth but does not alter competitive exclusion between specialist and generalist species
Source: PLoS One. 2025 Dec 17;20(12):e0328189. doi: 10.1371/journal.pone.0328189 (PMC12711014; doi:10.1371/journal.pone.0328189)
Supplement: S1 Table — (DOCX) [file pone.0328189.s001.docx]

Supporting Information file

| Table S1. Polynomial regression coefficients for aphid population dynamics models. | | | | | |
| --- | --- | --- | --- | --- | --- |
| Group | Term | Estimate | Std.Error | t.value | p.value |
| 1 | (Intercept) | 36.1563 | 2.6557 | 13.6173 | < 0.001 |
| 1 | day | 4.3284 | 26.0152 | 0.1664 | 0.868 |
| 1 | day^2^ | -142.4589 | 26.0152 | -5.4760 | < 0.001 |
| 2 | (Intercept) | 62.1042 | 5.1122 | 12.1483 | < 0.001 |
| 2 | day | 32.1257 | 50.0888 | 0.6414 | 0.523 |
| 2 | day^2^ | -376.7684 | 50.0888 | -7.5220 | < 0.001 |
| 3 | (Intercept) | 15.4479 | 1.7096 | 9.0361 | < 0.001 |
| 3 | day | -43.1184 | 16.7503 | -2.5742 | 0.012 |
| 3 | day^2^ | -59.2728 | 16.7503 | -3.5386 | 0.001 |
| 4 | (Intercept) | 45.3854 | 3.8444 | 11.8055 | < 0.001 |
| 4 | day | -100.3956 | 37.6676 | -2.6653 | 0.009 |
| 4 | day^2^ | -324.0100 | 37.6676 | -8.6018 | < 0.001 |
| 5 | (Intercept) | 12.0688 | 0.9398 | 12.8412 | < 0.001 |
| 5 | day | -82.4136 | 11.8882 | -6.9324 | < 0.001 |
| 5 | day^2^ | -59.7620 | 11.8882 | -5.0270 | < 0.001 |
| 6 | (Intercept) | 40.1813 | 3.3729 | 11.9130 | < 0.001 |
| 6 | day | 185.6215 | 42.6640 | 4.3508 | < 0.001 |
| 6 | day^2^ | -253.7219 | 42.6640 | -5.9467 | < 0.001 |
| 7 | (Intercept) | 10.6063 | 1.0463 | 10.1365 | < 0.001 |
| 7 | day | -96.9224 | 13.2352 | -7.3231 | < 0.001 |
| 7 | day^2^ | -22.4563 | 13.2352 | -1.6967 | 0.092 |
| 8 | (Intercept) | 47.0125 | 3.0064 | 15.6377 | < 0.001 |
| 8 | day | -8.2491 | 38.0278 | -0.2169 | 0.829 |
| 8 | day^2^ | -444.8575 | 38.0278 | -11.6982 | < 0.001 |
| This table presents the detailed statistical output for the quadratic regression models (y = ax² + bx + c) fitted to aphid population data over time, where y represents population size and x represents time in days. Group numbers correspond to treatments described in Table 1 of the main text. | | | | | |
