# Supplementary material for: Fertilization reduces aphid population growth but does not alter competitive exclusion between specialist and generalist species
Source: PLoS One. 2025 Dec 17;20(12):e0328189. doi: 10.1371/journal.pone.0328189 (PMC12711014; doi:10.1371/journal.pone.0328189)
Supplement: Attachment — Submitted filename: RL_Nakatani.docx [file pone.0328189.s004.docx]

Dear Dr. Saga,

Thank you for submitting your manuscript to PLOS ONE. After careful consideration, we feel that it has merit but does not fully meet PLOS ONE’s publication criteria as it currently stands. Therefore, we invite you to submit a revised version of the manuscript that addresses the points raised during the review process.

==============================

Both reviewers concur that the study holds great merit and relevance, and the overall structure of the manuscript is satisfactory and generally understandable as well as relevant to a wide potential readership

The main issues that need addressing are, broadly, as follows:

Literature review in Introduction appears too sketchy and narrow for such a cutting edge topic in modern agronomy with increasing pressure of pest upsurge against crop production. For example, in Line 97-102, the authors state that “Many studies have examined how aphid performance is influenced by plant quality ….” And then go ahead to list only 2 such citations. There is a vast volume of recent literature on this subject from which the authors may draw, to help place their research into a suitable gap-niche (i.e. justification for the study)

L99-124: We have substantially expanded the Introduction’s literature review to reflect the breadth of work on nutrient–aphid interactions and competitive dynamics. We added recent syntheses and field studies (e.g., Abdala-Roberts 2019; Aguilera 2021; Lescano 2022; Åhman 2019; Hamann 2021) and clarified the study gap: how host-plant nutrient status changes the **magnitude and direction of interspecific competition**, especially between a specialist and a generalist aphid.

The study’s aims are quite clear, and the hypotheses are very well articulated and backed with sound theoretical foundations that justify them, but these theoretical justifications need at least 2 relevant citations

L133-144: We added theoretical justifications with relevant citations in the Introduction. Specifically, we now ground our hypotheses in modern coexistence and resource‐competition theory (Chesson 2000; HilleRisLambers et al. 2012; Barabás et al. 2018) and in nutrient-driven bottom-up effects in herbivore–plant systems (Ali & Agrawal 2012; Abdala-Roberts et al. 2019).

Broaden the range of responses by both plants and aphids to perceived effects of fertilization (additional to counts and biomass measurements)

- We agree that additional physiological or chemical measurements could deepen the interpretation of fertilization effects. However, our experiment was designed to focus on demographic outcomes (aphid abundance and plant biomass) which directly reflect resource competition strength.
- L496-505: We have added a note in the Discussion acknowledging this limitation and emphasizing that future studies should include physiological and biochemical traits to clarify the underlying mechanisms.

Explain clearly in response to Reviewer #1 to what extent your experimental protocol and results obtained may be practically applicable in real life/field situations outside the controlled laboratory condictions

- We agree that direct extrapolation to field conditions should be made cautiously. The experiment was conducted under controlled conditions to isolate nutrient and competition effects.
- L512-520: We now clarify in the Discussion that while absolute values may differ in the field, the direction of responses we observed aligns with field studies (e.g., Staley et al., 2011; Aguilera et al., 2021) and provides a theoretical basis for future field validations.

The provided statement of experimental strategy for minimizing aphid movement inter-treatment (e.g. clipping wings) does not seem to suffice. Unless legs were also clipped, can’t the insects still disperse around freely?

- We apologize for the insufficient description. In addition to removing wings from alate individuals, all pots were placed inside separate plastic trays filled with a thin layer of water to prevent aphids from moving between pots. This water barrier effectively restricted movement while avoiding any harm to the insects.
- L185-190: We have clarified this procedure in the revised Methods section.

Experimental method protocol needs much more detail, including reference to standard procedures, authority citations and instrumental/material detailing to support validation and repeatability/replication

- We appreciate this suggestion.
- L181-182, 197-199: We revised Materials and Methods to add procedural detail and authoritative citations. We now cite Moritsu (1983) for species identification and Staley et al. (2011) for experimental setup and census schedule. These changes improve transparency and reproducibility

Provide elaborate references/citations for the various statistical tools/equations/theories adopted for analyses

- L213-237: We have added authoritative citations supporting each statistical method, including polynomial regression and ANOVA/Tukey (Zar, 2014; Fox & Weisberg, 2019), survival analysis (Kaplan & Meier, 1958; Mantel, 1966), and multiple-comparison control (Benjamini & Hochberg, 1995). These additions ensure clarity, replicability, and methodological validation.

Provide at least one Figure showing a color-separated images of the study species of aphids

We have added images of both aphid species to Figure 1 (panels a and b).

Nickson Erick Otieno, PhD

Academic Editor

PLOS ONE

Journal Requirements:

When submitting your revision, we need you to address these additional requirements.

1.Please ensure that your manuscript meets PLOS ONE's style requirements, including those for file naming. The PLOS ONE style templates can be found at

https://journals.plos.org/plosone/s/file?id=wjVg/PLOSOne_formatting_sample_main_body.pdf and

<https://journals.plos.org/plosone/s/file?id=ba62/PLOSOne_formatting_sample_title_authors_affiliations.pdf>

We have revised the manuscript to comply with PLOS ONE's style requirements.

2. We note that the grant information you provided in the ‘Funding Information’ and ‘Financial Disclosure’ sections do not match.

When you resubmit, please ensure that you provide the correct grant numbers for the awards you received for your study in the ‘Funding Information’ section.

Thank you for pointing this out. The research was partially supported by internal funds from Kobe University that were provided through a JST (Japan Science and Technology Agency) program to support high school research collaboration. This support does not have a specific grant number.

3. Thank you for stating the following financial disclosure:

“AN received funding from the JST Global Science Campus ROOT Program.”

Please state what role the funders took in the study. If the funders had no role, please state: "The funders had no role in study design, data collection and analysis, decision to publish, or preparation of the manuscript."

If this statement is not correct you must amend it as needed.

Please include this amended Role of Funder statement in your cover letter; we will change the online submission form on your behalf.

The funders had no role in study design, data collection and analysis, decision to publish, or preparation of the manuscript.

4. When completing the data availability statement of the submission form, you indicated that you will make your data available on acceptance. We strongly recommend all authors decide on a data sharing plan before acceptance, as the process can be lengthy and hold up publication timelines. Please note that, though access restrictions are acceptable now, your entire data will need to be made freely accessible if your manuscript is accepted for publication. This policy applies to all data except where public deposition would breach compliance with the protocol approved by your research ethics board. If you are unable to adhere to our open data policy, please kindly revise your statement to explain your reasoning and we will seek the editor's input on an exemption. Please be assured that, once you have provided your new statement, the assessment of your exemption will not hold up the peer review process.

Thank you for your advice. We will prepare and implement our data sharing plan immediately after submitting the revised manuscript.

5. Please include your full ethics statement in the ‘Methods’ section of your manuscript file. In your statement, please include the full name of the IRB or ethics committee who approved or waived your study, as well as whether or not you obtained informed written or verbal consent. If consent was waived for your study, please include this information in your statement as well.

L241-245: We have added an ethics statement in the Materials and Methods section. According to the Regulations for Animal Experimentation and the Ethics Guidelines for Research Involving Human Subjects of Kobe University, ethical review was not required because the study involved only plants and invertebrate insects. Field sampling was conducted on private land owned by the first author’s family with permission.

6. Please include captions for your Supporting Information files at the end of your manuscript, and update any in-text citations to match accordingly. Please see our Supporting Information guidelines for more information: http://journals.plos.org/plosone/s/supporting-information.

Done. We have added captions for all Supporting Information files at the end of the manuscript and updated in-text citations accordingly.

7. If the reviewer comments include a recommendation to cite specific previously published works, please review and evaluate these publications to determine whether they are relevant and should be cited. There is no requirement to cite these works unless the editor has indicated otherwise.

Done. We have reviewed the recommended publications and cited those relevant to our study.

Additional Editor Comments:

Each of the reviewers has, in addition to the general comments and statements of concern or suggested changes, also offered additional specific comments in the article PDF which is attached to the authors in the submission platform. The authors must carefully access these, address each of the comments and, in resubmitting a thoroughly revised manuscript, include a separate document detailing how each of these issues has been addressed point by point, or a robust rebuttal as to why they disagree with suggested changes

The formatting of the document has not adhered to the guidelines of PLOS ONE. In assessing the revised manuscript, the strict compliance to formatting guidelines (structure, font sizes and styles as well as arrangement of the various component parts) will be the first criteria before the paper can progress any further, failure to which it will have to be rejected outright. For instance, in-text citation style uses numbers, not author-and-year; they must also be enclosed in square brackets, and listed in the corresponding number orders under References at the end.

Tables and their captions must be immediately below paragraphs they are first mentioned in;

Figure captions must be in the positions below paragraphs they are first mentioned in

Refer to submission guidelines for strict compliance will all requirements

We have also revised the manuscript to fully comply with PLOS ONE formatting guidelines, including citation style, reference numbering, and placement of tables and figures.

Reviewers' comments:

Reviewer #1: Dear Authors,

Thank you for submitting your manuscript to Plus One. The study addresses a valuable question about how fertilization shapes aphid performance and interspecific competition on pea plants. While your work has merit, the manuscript, in its current format, does not meet the journal’s standards for publication. Several important methodological and interpretive issues need to be addressed, and I have outlined them below with guidance to help you prepare a stronger revision. A separate document with detailed, itemized suggestions is attached.

Main issues to consider and revise:

1- Trait scope and depth:

- The current trait set (aphid counts and plant biomass) is too limited to robustly infer mechanisms of plant response to aphid infestation and to establish the influence of fertilization on competitive outcomes.

- Consider adding additional plant responses (e.g., chlorophyll content, photosynthetic rate, stomatal conductance, tissue nutrient concentrations, defensive metabolites) and aphid performance metrics (e.g., reproduction rate, development time, adult longevity, honeydew production) to better capture trophic interactions and tolerance mechanisms.

We agree that expanding plant and aphid trait measurements (e.g., chlorophyll, photosynthesis, tissue nutrients, defensive metabolites, reproduction and longevity metrics) would strengthen mechanistic inference. However, these variables were not measured in the present experiment, which was designed as a first step to isolate fertilization effects on population trajectories under controlled conditions.

L496-505: We now acknowledge this explicitly as a limitation and outline it as a priority for future work.

2- Proof of fertilization effect on species-specific aphid numbers:

Clarify how fertilization differentially affected the two aphid species. Provide explicit experimental design details and statistical tests that support the claim that changes in aphid counts by species are attributable to fertilizer treatment rather than other factors.

348-360; To avoid confounding by interspecific interactions, we analyzed only single-species treatments using two-way ANOVA (Species × Fertilizer) on AUC, peak, and end-point counts (log-transformed). Fertilization significantly reduced *M. crassicauda* performance but not *A. craccivora*. The Species × Fertilizer interaction was significant for end-point counts (F₍₁,₂₀₎ = 8.94, *p* = 0.0072), confirming species-specific effects. Methods and results have been added to the revised manuscript and Figure 2.

3- Containment and movement between pots:

Describe how you prevented aphid movement between pots (physical separation, barriers, spatial layout, or any measures to minimize cross-transfer). If movement was possible, discuss its potential impact on results and how it was mitigated or accounted for in analyses.

L185-190: We have added details on containment and spacing. Each pot was placed in an individual tray (24 × 17 × 5 cm) with a 2 cm water layer acting as a moat, and pots were spaced at least 10 cm apart (Fig 1c). Alate wings were removed upon detection. These measures effectively prevented aphid movement between pots.

4- Plant tolerance assessment

Biomass alone is insufficient to characterize plant tolerance to herbivory. Include additional indicators (growth rates, tissue allocation patterns, physiological stress indicators, biochemical markers) to support conclusions about tolerance under different nutrient regimes.

L496-505; We agree that biomass alone is insufficient to assess tolerance. As these measurements were not collected in the present study, we now acknowledge this as a limitation and note a plan to include tolerance indicators (growth rate, tissue allocation, physiological/biochemical stress markers) in future work.

5- Repetition and clarity regarding trait choices

There are repeated points about the limited trait set. Consider consolidating these into a single, clear rationale for expanding trait measurements and present a concrete plan for the expanded suite of traits.

L496-505; We consolidated all trait-related limitations and plans into one clear paragraph and removed repetition. No additional trait paragraphs were added.

6- Methods: measurement references

The Materials and Methods section should include explicit references or protocols for all measurement techniques. Provide enough detail for replication, including instrument models, measurement conditions, sampling timing, and data processing steps.

We added detailed methodological references rather than generic protocol citations. Specifically, we now (1) cite Moritsu (1982) for aphid identification, (2) reference Staley et al. (2011) for experimental design rationale, and (3) include key statistical references (Fox & Weisberg, 2019; Kaplan & Meier, 1958; Mantel, 1966; Benjamini & Hochberg, 1995). These revisions ensure reproducibility and proper attribution of established methods.

7- Language and presentation

Consider a careful language edit to improve clarity and conciseness. Clear, precise phrasing will help readers understand the experimental design, results, and interpretation.

We have revised the manuscript throughout to improve clarity and conciseness. All edits are highlighted in green.

I hope you will consider a substantial revision.

Specific comments

Abstract: The results presented in the abstract are not sufficient.

Thank you for the guidance. We revised the Abstract to report key quantitative outcomes (representative means ± SD, time to exclusion, and plant-survival result), making the results sufficiently explicit.

Introduction: Most of the references used in the introduction are old and should be rewritten with newer references.

L108-109: So old!

Thank you for the comment. We have reviewed recent literature and updated the Introduction with appropriate and up-to-date references.

L165-170: How did you make sure the aphids didn't move to neighboring pots?

We have added explicit details on containment (L182-187) and included a photograph of the experimental setup in Figure 1c showing the water moat system that prevented aphid movement between pots.

L190-191: Please add reference

L214-215: We added a reference.

Results: The traits evaluated in this study are very limited and it is strongly recommended that other traits be added, especially those related to plant response to aphid infestation.

We appreciate the suggestion. The study focused on population dynamics and whole-plant responses, which were sufficient to address our research question. Additional physiological traits are indeed valuable but beyond the current study’s scope.

Results, Population dynamics differed between fertilizer treatments:

How do you prove the changes in the number of aphids by species on the plant as a result of fertilization? In other words, how can you be sure that the changes are definitely due to the presence or absence of fertilizer. Specify this clearly in the Materials and Methods.

L228-234, 271-282: Thank you for the comment. We have clarified in Materials and Methods how fertilization effects were identified: we manipulated only fertilization in single-species assays with random assignment and identical starting densities, controlled all other conditions, and tested Species × Fertilizer using two-way ANOVA on pre-specified per-pot summaries. This isolates fertilizer as the cause of the observed species-specific changes.

Discussion: Host Plant Responses and Implications

The degree of tolerance of a plant to herbivory cannot be determined by evaluating only one trait (biomass). There are many growth, physiological, and biochemical indicators in this regard that the authors did not consider.

L31-32, L146-152, L209-211, L328-329, L452-456, L478-485:

Thank you for this point. We are not claiming to quantify physiological tolerance. We revised the Abstract, Introduction, Methods, Results, and Discussion to clarify that plant response was assessed using whole-plant indicators (survival time, biomass change), not tolerance traits; we also softened wording about tolerance/resistance and framed such ideas as hypotheses.

Reviewer #2: The paper is well written but it deals only with a laboratory experiment lasted only 30 days. I think replication is needed to confirm results. Also, Figure 1 is not very legible and difficult to interpret.

We appreciate this comment. We agree that our study is a single, 30-day laboratory experiment.

L516-519: We now state this limitation explicitly and note plans to replicate the assay across time (independent runs) and settings (greenhouse/field with natural enemies) to confirm robustness.

Figure 1 has been revised to improve visual clarity, with labeling adjusted. In addition, we added Figure 2 to summarize per-pot metrics (AUC), which helps readers interpret species-specific fertilizer effects more easily.

Specific comment

Please indicate what kind of fertilisation (Nitrogen, Potassium.....)?

L26: We have clarified the type and composition of the fertilizer in the Abstract.
